# The impact of multidisciplinary collaborative bundled care on analgesia and sedation in ICU patients with endotracheal intubation

**DOI:** 10.1097/MD.0000000000040901

**Published:** 2024-12-20

**Authors:** Xiaohui Wu, Longping Yu

**Affiliations:** a The First Affiliated Hospital of Soochow University, Suzhou, China.

**Keywords:** analgesia and sedation, cough strength, delirium, mechanical ventilation, multidisciplinary collaboration

## Abstract

**Background:**

Effective analgesia and sedation management play a crucial role in reducing the intensity of coughing in patients with endotracheal intubation and improving clinical outcomes. However, current approaches are predominantly singular and lack comprehensive management strategies based on multidisciplinary collaboration. This study aims to evaluate the impact of multidisciplinary collaborative bundled care on analgesia and sedation in intensive care unit (ICU) patients with endotracheal intubation, providing evidence to inform clinical practice.

**Methods:**

Seventy ICU patients were enrolled with endotracheal intubation, admitted between January and August 2024. They were divided into a control group (n = 35) receiving routine care (admitted from January to April) and an intervention group (n = 35) receiving multidisciplinary collaborative bundled care (admitted from May to August). Outcomes measured included physical restraint use, analgesic dosage, cough strength, delirium incidence, and duration of mechanical ventilation.

**Results:**

The intervention group had significantly lower doses of remifentanil (8.37 ± 1.50 mg) and midazolam (21.43 ± 3.74 mg) compared to the control group (9.92 ± 1.58 and 31.12 ± 7.89 mg; *P* < .05). The incidence of delirium was also lower in the intervention group (11.4%) than in the control group (31.4%; *P* < .05). Delirium onset was delayed in the intervention group (3.02 ± 1.05 days) compared to the control group (2.58 ± 0.79 days), and its duration was shorter (3.43 ± 1.74 vs 5.12 ± 1.89 days; *P* < .05). Additionally, physical restraints were significantly lower in the intervention group (22.9% vs 45.7%; *P* < .05). Cough strength was notably higher in the intervention group (4.74 ± 0.82 vs 3.36 ± 0.76; *P* < .05), and the duration of mechanical ventilation was reduced (4.77 ± 1.42 vs 5.92 ± 1.66 days; *P* < .05).

**Conclusion:**

Multidisciplinary collaborative bundled care improves sedation and analgesia outcomes in ICU patients with endotracheal intubation, reducing medication dosage, incidence of delirium, physical restraint use, and duration of mechanical ventilation while enhancing cough strength.

## 1. Introduction

Mechanical ventilation by endotracheal intubation is an important means of life support for patients with acute and critical illnesses, and approximately 40% of intensive care unit (ICU) patients require mechanical ventilation by endotracheal intubation to maintain respiratory function.^[1]^ Due to the specificity of the treatment, mechanical ventilation by tracheal intubation is often associated with varying degrees of physiological–psychological stress responses in patients, causing physiological and psychological discomfort.^[2]^ In addition, ICU patients with endotracheal intubation are usually in either a fully immobilized state (where the patient is completely immobilized and remains in a fixed position) or a semi-immobilized state (which allows limited movement within a restricted area). This results in reduced physiological and airway function. The need for multiple tubes further reduces the patient’s physical and psychological comfort, increasing the likelihood of adverse events, prolonging ICU stays, and increasing the risk of poor outcomes. Therefore, in recent years, the role of analgesic and sedative interventions in the clinical management of endotracheal intubated patients in the ICU has been emphasized to reduce the adverse effects of physiological and psychological discomfort on patients.

National and international expert consensus or guidelines on analgesia and analgesic treatment in the adult ICU also recommend analgesia and sedation as routine treatment for mechanically ventilated ICU patients.^[3,4]^ Analgesia and sedation can not only increase patient comfort, reduce anxiety, and improve respiration but also help to reduce the patient’s stress response and oxygen consumption, correct tissue hypoxia, and thus reduce damage to organ function, but the phenomena of over-sedation and under-sedation must be avoided.^[5]^ Insufficient sedation and analgesia will lead to mechanical ventilation patients with accelerated heart rate, elevated blood pressure, human-machine confrontation, and other adverse consequences. In contrast, excessive sedation and analgesia may lead to drug accumulation in the body, impairing liver, kidney function, and central nervous system function, resulting in delirium, respiratory depression, coughing, and sputum production of the patient, and even increasing the risk of lung infection.^[6]^ Some studies have shown that the incidence of delirium in tracheal intubated patients in the ICU is currently as high as 65% to 80%,^[7]^ and the incidence of excessive sedation is also between 10% and 25%.^[8]^ The occurrence of both delirium and diminished cough strength affects the time to extubation, leading to a further increase in the duration of mechanical ventilation and a corresponding increase in the incidence of mechanical ventilation-related complications and mortality.^[9]^ Therefore, rational analgesic and sedation management is essential to reduce worsening coughs and improve clinical outcomes in tracheal intubation patients. Although there are many studies on managing analgesia and sedation in patients with tracheal intubation, most of the measures are relatively simple and lack a comprehensive management strategy based on multidisciplinary collaboration.

Multidisciplinary teamwork (MDT) was first developed by a group of medical experts in the United States in the 1960s and 1970s and involves a team of professionals with different disciplinary backgrounds and senior specialists. The MDT team includes doctors, nurses, rehabilitation therapists, psychological counselors, etc. It discusses in the form of meetings/consultations, focusing on the patient’s condition problems, collaborating and sharing information,^[10]^ and integrating healthcare resources by forming a working group and relying on multidisciplinary teams to provide patients with the most effective diagnosis, treatment, and care plan and to promote early recovery of patients.^[11]^ In recent years, the MDT model has been widely adopted in the United States, Australia, and other countries in the areas of postoperative breast cancer, senior care, and accelerated recovery surgery. It is internationally recognized as the most effective method of disease management. The MDT model was also promoted in the assessment indicators of China’s Action Plan for Further Improvement of Medical Services (2018–2020) in 2018. Relevant studies at home and abroad have also confirmed that the development of analgesia and sedation management plans for mechanically ventilated patients in ICUs under the MDT model can achieve cross-collaboration and resource complementation among different disciplines, optimize clinical treatment and care plans, and improve patient prognosis.^[12]^ However, the number of people and the disciplinary composition of MDT teams in different hospitals differ. Therefore, in this study, we propose to create a multidisciplinary team based on the actual clinical needs of mechanically ventilated patients to manage patients’ analgesia and sedation based on the MDT model to improve patient’s clinical outcomes.

In 2001, the Institute for Health Care Improvement proposed bundled care. This nursing model usually consists of several evidence-based treatments and nursing measures to prevent and manage patients’ health conditions and treat difficult-to-treat clinical diseases.^[13]^ The sedation-analgesia bundle strategy is an innovative management strategy, namely the ABCDE bundle, which includes daily awakening, breathing synchronization, sedative and analgesic drug selection, delirium monitoring and treatment, and early exercise.^[14]^ Related studies have shown that applying the sedation-analgesia bundle strategy can effectively prevent complications such as ventilator-associated pneumonia, ICU-acquired delirium, and ICU-acquired myasthenia in patients with endotracheal intubation and can improve patient prognosis.^[15]^ In addition, compared with conventional sedation and analgesia care measures, sedation and analgesia bundled care measures can reduce the amount of analgesic and sedative drugs used in mechanically ventilated patients, reduce drug-induced iatrogenic coma, shorten the duration of mechanical ventilation and hospitalization, and reduce the incidence and mortality of acquired delirium.^[16]^ This study integrated the 2 models based on the positive effects of the MDT model and analgesia and sedation bundled care on endotracheal intubation patients. Based on multidisciplinary collaboration, it developed analgesia and sedation bundled care measures for endotracheal intubation patients. It applied them clinically, aiming to provide a clinical reference for the analgesia and sedation management of clinical endotracheal intubation patients, improve the cough intensity of patients, reduce the incidence of delirium, shorten the duration of mechanical ventilation, and improve the prognosis of patients.

## 2. Objects and methods

### 2.1. Study subjects

Patients admitted to the ICU of our hospital from January 2024 to August 2024 who underwent endotracheal intubation and mechanical ventilation were selected as study subjects.^[4]^ Inclusion criteria: age ≥18 years; underwent endotracheal intubation with a mechanical ventilation duration ≥24 hours; indicated for sedation and analgesia therapy; no mobility impairment prior to admission, with normal hearing, cognitive function, and communication abilities; patients and their families were informed about the purpose of the study and provided consent to participate. Exclusion criteria: loss of cough reflex upon admission; comorbid psychiatric disorders; history of alcohol abuse or substance use; comorbid neuromuscular diseases or limb mobility impairments; withdrawal from the study for any reason. The study was reviewed and approved by the Ethics Committee of the first affiliated hospital of Soochow University (2024-615), and informed consent was obtained from all patients included in the study.

#### 2.1.1. Sample size calculation

The sample size was calculated using the 2-sample mean comparison formula: n_1_ = n_2_={(Z*ɑ*/2 + Z*β*) × *σ*/*δ*}². Assuming a significance level of *α* = 0.05 and a power of 1 − *β* = 0.9, the corresponding Z-values are Z*α*/2 = 1.96 and Z*β* = 1.282 for a 2-tailed test. Based on pilot study results, the standard deviation (*σ*) was set at 4.02, and the effect size (*δ*) was set at 3.49. Substituting these values into the formula yields n_1_ = n_2_ = 28, for a total sample size of 56. Considering a 10% attrition rate, the final sample size was determined to be 70 participants.

#### 2.1.2. Randomization and blinding

Simple randomization and double-blinding were employed. Pseudo-random numbers with a uniform distribution were generated using SPSS 21.0 software and ranked. Numbers 1 to 35 were assigned to group A, and numbers 36 to 70 to group B. These numbers were sealed in opaque envelopes, and the patients’ family members randomly selected an envelope. Participants who drew numbers 1 to 35 were allocated to group A (control group), and those who drew numbers 36 to 70 were assigned to group B (intervention group). Both groups included 35 participants.

### 2.2. Methods

#### 2.2.1. Formation of the multidisciplinary collaborative team

First, a multidisciplinary collaborative team is established, led by the ICU charge nurse. Team members include the ICU charge nurse, ICU consultant, respiratory therapist, psychological counselor, dietician, and neurologist. All members of the team have intermediate or senior professional qualifications and at least 5 years of experience in the ICU. The ICU consultant is the team leader and is responsible for formulating the sedation and analgesia treatment plan and managing the use of medications. The senior ICU nurse acts as deputy team leader, overseeing team training and quality control to ensure standardized implementation of the plan.

#### 2.2.2. Implementation process

The control group implements routine analgesic and soothing care, which includes: closely observing changes in the patient’s vital signs; evaluating patients’ level of consciousness and the degree of sedation and analgesia once an hour, and reporting the results to the doctor in charge, and adjusting the drug administration program according to the doctor’s instructions; evaluating changes in patients’ muscle strength every shift; turning patients regularly and aspirating sputum on time and as needed; perfecting tube care; doing a good job of skin care, psychological care and other basic nursing work.

The intervention group implemented a multidisciplinary collaborative approach to analgesia and sedation through bundled care. The process was as follows: first, the charge nurse of the ICU coordinated the professional training of the team members. The training included the importance, procedural steps, detailed implementation methods, precautions, and the use and interpretation of various assessment scales related to bundled care. The training was delivered through a combination of focused slide presentations and practical bedside sessions. Participants then underwent both theoretical and practical assessments, and only those who passed were eligible to participate in the implementation of the study. Multidisciplinary bundle care interventions were initiated 24 hours after mechanical ventilation in intubated patients, as their condition was relatively stable at this time, allowing them to better adapt to the interventions. Details of the interventions are provided in Table 1. The intervention period in this study was defined as the duration from the initiation of mechanical ventilation with sedation and analgesia to the complete cessation of these medications and successful weaning from the ventilator.

**Table 1:** Bundled nursing measures for analgesia and sedation in patients with endotracheal intubation.

Nursing measures	Participants	Implementation methods
Daily wake-up test^[17]^	Primary nurse, attending physician, respiratory therapist	The nurse responsible, the attending physician, and the respiratory therapist assess the safety of the daily arousal test. All sedatives and analgesics are stopped at 9 am each day for those who meet the criteria, and the arousal test is performed. The test is successful if the patient is awake and can answer 3 to 4 simple questions. During the test, the patient’s vital signs are closely monitored. If the patient is uncomfortable or agitated, the test is stopped immediately, and appropriate treatment is given.
Spontaneous breathing test^[18]^	Primary nurse, attending physician, respiratory therapist	Suppose the patient’s arousal test is successful. In that case, the nurse in charge, in the presence of the attending physician, will assist the respiratory therapist in assessing the safety of the spontaneous breathing test, administer the spontaneous breathing test to those who are eligible, observe the patient’s vital signs and level of consciousness, and at the same time record the ventilator’s operating status, mode, parameters, and human-computer coordination. If the patient experiences discomfort during the test, the test should be stopped immediately, and appropriate treatment should be given according to medical advice.
Sedative and analgesic drug use	Primary nurse and attending physician	Remifentanil and midazolam were selected as first-line analgesics and sedatives according to the guidelines for sedation and analgesia in adult ICUs. The nurses in charge assessed the patient’s pain, analgesia, and sedation status using the CPOT^[19]^ and the RASS^[20]^ at the end of each shift (08:00 and 20:00). The CPOT total score was 8, with a target score of ≤2; the RASS target score was −2 to 1. According to the target scores of the above 2 scales, the dosages of sedatives and analgesics and the speed of the microinjection pump were adjusted promptly according to the physician’s instructions. The responsible nurses operated strictly and recorded the type, dosage, infusion speed, etc, of the drugs until the drugs were stopped.
Delirium monitoring	Primary nurse, attending physician, neurologist	When the patient’s RASS score is ≤−3, the attending physician and primary nurse use the CAM-ICU to assess for delirium. If both results are positive, the patient is treated for delirium. If there was disagreement between the 2 results, the outcome was assessed by a neurologist.
Physical restraints	Primary nurse and attending physician	Together with the patient’s actual situation, the medical staff will evaluate and decide whether to use physical restraints. For patients requiring physical restraint, the nurse in charge will apply graduated physical restraint according to the restraint decision wheel and the Chinese Nursing Association’s group standard “Physical Restraint Nursing for Inpatients.” During the application of physical restraint, the nurse in charge will perform dynamic assessments, apply and release the restraints every 2 h, and observe the skin and blood circulation of the restrained area every 2 h. For patients who meet the analgesic and sedative standards, physical restraints will be removed as soon as possible.
Early mobilization	Primary nurse and rehabilitation therapist	For patients with no contraindications to exercise, the nurse in charge and the rehabilitation therapist work together to help patients perform early activities twice daily (10:00 am and 5:00 pm). The early activity program is divided into 5 phases.^[21]^ The activity program should follow the principle of gradual progress and patient tolerance, and the activity program should be adjusted at any time according to the patient’s muscle strength and RASS score. The first stage is active and passive movement of the upper limbs, chest, and lower limbs; the second stage is voluntary sideways movement based on the previous stage; the third stage is sitting up in bed based on the previous stage; the fourth stage is sitting beside the bed with both feet on the floor and both hands supported based on the previous stage; the fifth stage is getting out of bed based on the previous stage.
Early nutrition	Attending physician, primary nurse, and nutrition therapist	After assessing the patient’s condition, the attending physician, nurse, and nutritionist will formulate an early nutrition support plan. Enteral nutrition should be provided to patients who can receive early enteral nutrition. Nursing staff should provide appropriate care during enteral nutrition support: raise the head of the bed by 30° to 45°, observe the patient’s complexion and whether there are symptoms such as vomiting, aspiration, choking, and dyspnea. The volume of enteral nutrition solution increases from small to large, and the rate increases from slow to fast. Enteral nutrition is given through a nasojejunal or gastric tube, and an electronic feeding pump is used to ensure a steady and slow infusion. During the infusion, a heater is used to maintain the temperature of the nutritional solution between 37°C and 40°C. Suppose the patient experiences discomfort during the enteral nutrition infusion, such as abdominal distension, pain, or diarrhea. In that case, the drip rate should be slowed, the infusion should be stopped immediately, and the physician should be notified immediately for timely treatment; for those intolerant to enteral nutrition, appropriate strategies should be used to improve tolerance as much as possible.
Psychological care	Primary nurse and psychological counselor	Nurses and psychological counselors provide psychological support to conscious patients, explain the necessity and temporary nature of endotracheal intubation, the importance of early activity and nutrition, arrange daily video calls with family members, play soothing music for patients, and reduce their tension and anxiety.

CAM = confusion assessment method, ICU = Intensive care unit, RASS = Richmond Agitation-Sedation Scale.

#### 2.2.3. Data collection methods

General patient information, including age, sex, diagnosis, etc, is mainly obtained from medical records. Amount of analgesic and sedative drugs used: comparison of the total amount of drugs used, such as remifentanil and midazolam, on the third day of the intervention between the 2 groups. Incidence of delirium: including number of cases of delirium, time of onset, and duration of delirium. Restraint use and mechanical ventilation time: check the patient’s restraint use and mechanical ventilation time from the physician’s order and nursing record. Assessment of cough strength^[17]^: in the presence of the attending physician, the responsible nurse and respiratory therapist assisted the ultrasound team members in assessing the patient’s cough strength. The principle of this method is to assess the patient’s cough strength by measuring the amplitude of diaphragmatic movement during deep breathing under M-mode ultrasound. Procedure: the patient lies in the supine position. The members of the ultrasound team place the ultrasound probe on the abdominal wall under the patient’s right ribs, between the axillary line and the sternal line on the longitudinal scan plane, using the liver as an acoustic window.^[18]^ The angle of the probe is adjusted so that the ultrasound beam is perpendicular to the posterior 1/3 of the right diaphragm. The respiratory therapist instructs the patient to perform 3 maximal and effective spontaneous coughs. The ultrasound team members measure the diaphragm excursion amplitude and take the average of the 3 times. The greater the diaphragm excursion amplitude, the stronger the patient’s cough.^[19]^

### 2.3. Statistical analysis

The data were processed using SPSS 21.0 statistical software. Quantitative data following normal distribution were expressed as mean ± standard deviation and analyzed using an independent sample *t* test. Qualitative data conforming to a normal distribution were presented as percentages (%) and assessed using the χ² test. Non-normally distributed data were analyzed using the rank-sum test. The test level was *P* < .05 for statistical differences.

## 3. Results

### 3.1. Comparison of general characteristics between the 2 groups

There were no statistically significant differences between the 2 groups in terms of age, gender, or type of mechanical ventilation disease, indicating comparability (*P* > .05). Details are provided in Table 2.

**Table 2:** Comparison of general information between the 2 groups of patients.

Group	Gender		Age (yr)	Types of mechanically ventilated disease			
	Male	Female		Respiratory failure	Postoperative	Circulatory disease	Other
Intervention group	21 (60.0%)	64.20 ± 19.72	64.20 ± 19.72	16 (45.7%)	9 (25.7%)	6 (17.1%)	4 (11.4%)
Control group	20 (57.1%)	58.96 ± 17.85	58.96 ± 17.85	14 (40.0%)	10 (28.6%)	8 (22.9%)	3 (8.6%)
*t*/χ^2^ value	0.059		−1.055	0.615			
*P* value	.808		.296	.893			

### 3.2. Analgesic and sedative drug usage

The intervention group required significantly less remifentanil and midazolam than the control group over 3 days (*P* < .05). See Table 3 for details.

**Table 3:** Comparison of the amount of analgesic and sedative drugs used by the 2 groups of patients.

Group	Remifentanil (mg)	Midazolam (mg)
Intervention group	8.37 ± 1.50	21.43 ± 3.74
Control group	9.92 ± 1.58	31.12 ± 7.89
*t* value	−3.862	−6.345
*P* value	.000	.000

### 3.3. Incidence and characteristics of delirium

The intervention group exhibited a lower incidence of delirium, with a later onset and shorter duration compared to the control group. All differences were statistically significant (*P* < .05), as shown in Table 4.

**Table 4:** Comparison of the incidence of delirium between the 2 groups of patients.

Group	Incidence (%)	Delirium onset time/d	Delirium duration/d
Intervention group	11.4% (4/35)	3.02 ± 1.05	3.43 ± 1.74
Control group	31.4% (11/35)	2.58 ± 0.79	5.12 ± 1.89
*t*/χ^2^ value	4.158	2.954	−4.571
*P* value	.041	.000	.000

### 3.4. Physical restraint usage, cough intensity, and mechanical ventilation duration

The intervention group had a lower rate of physical restraint use, greater diaphragm excursion amplitude, and shorter mechanical ventilation duration than the control group, with statistically significant differences (*P* < .05). Refer to Table 5 for details.

**Table 5:** Comparison of the rate of use of physical restraints, cough intensity, and mechanical ventilation time between the 2 groups of patients.

Group	Physical restraint use rate (%)	Diaphragm excursion amplitude (cm)	Mechanical ventilation time (d)
Intervention group	8 (22.9%)	4.74 ± 0.82	4.77 ± 1.42
Control group	16 (45.7%)	3.36 ± 0.76	5.92 ± 1.66
*t* value	4.885	6.662	−2.886
*P* value	.027	.000	.005

## 4. Discussion

### 4.1. Analgesia and sedation bundled care based on multidisciplinary collaboration can reduce the use of analgesic and sedative drugs in patients with endotracheal intubation

The results of this study showed that the use of analgesic and sedative drugs was lower in the intervention group than in the control group, demonstrating that analgesic and sedative cluster care based on multidisciplinary collaboration can reduce the use of analgesic and sedative drugs in patients undergoing tracheal intubation. As part of the multidisciplinary intensive care, the attending physician, respiratory therapist, consultant, and ward nurse performed daily arousal and spontaneous breathing tests on patients to accurately record the time of patient weaning and reduce the use of drugs due to delayed weaning. First, the daily sedative infusion was suspended until the patient was awake or agitated, the patient’s level of consciousness was assessed, and the voluntary breathing test and cough strength measurement were performed to determine the patient’s extubation status. The dosage and rate of sedatives were adjusted according to the patient’s CPOT and RASS scores so that the patient could achieve good analgesia and sedation while reducing the sedative dosage to avoid the metabolic burden or inadequate sedation that may occur due to delayed weaning of the patient from the ventilator. Avoidance of adverse events such as detachment due to overdosage of sedatives, exacerbation of organ metabolic stress, or inadequate sedation. Second, psychological care in intensive care achieves the effect of non-pharmacological analgesia. Through psychological counseling by the psychological consultant, the video visit of family members, the playing of soothing music, and other measures, the patients are given care and encouragement to alleviate their negative emotions, such as anxiety and nervousness, and to achieve the effect of auxiliary analgesic and soothing treatment, thus reducing the amount of analgesic and sedative drugs used.

### 4.2. Analgesia and sedation bundled care based on multidisciplinary collaboration can reduce the incidence of delirium in patients with endotracheal intubation

The results of this study showed that the incidence of delirium in the intervention group was lower than that in the control group, the onset of delirium in the intervention group was later than that in the control group, and its duration was shorter than that in the control group, indicating that analgesic and sedative intensive care based on multidisciplinary collaboration can reduce the incidence and duration of delirium in patients undergoing endotracheal intubation, which is consistent with the findings of Negro et al.^[20]^ Delirium is an acute state of confusion, usually accompanied by cognitive dysfunction, decreased attention, impaired consciousness, and increased or decreased psychomotor activity. It has a high incidence in mechanically ventilated ICU patients. Some studies have shown that the occurrence of delirium in mechanically ventilated ICU patients is associated with sedation, which is an independent risk factor for the development of delirium, and the use of sedation increases the risk of ICU delirium by a factor of 2.78.^[21]^ Robinson and Vollmer’s^[22]^ study highlights that insufficient sedation can elevate the risk of delirium and contribute to issues such as restlessness, patient-ventilator dyssynchrony, and unintentional extubation. Further research demonstrates that the onset of delirium is linked to pain, which elevates catecholamine levels in the blood, reducing tissue perfusion.^[22]^ Additionally, stress-induced decreases in brain acetylcholine levels are implicated in delirium development.^[23]^ Consequently, appropriate analgesia and sedation are critical for preventing ICU delirium. In this study, multidisciplinary analgesia and sedation cluster-based care standardized the process of implementing interventions, the evaluation criteria, the implementation time of each step, and the operators, and the whole process brought together multiple disciplines such as doctors, nurses, rehabilitation therapists, respiratory therapists, nutrition therapists, neurologists, etc to make the entire process more standardized and standardized. Through timely assessment and adjustment, the multidisciplinary team could keep the level of analgesia and sedation to the standard and avoid under- or over-analgesia, thus reducing the incidence of delirium due to inappropriate analgesia and sedation. Second, early rehabilitation and early enteral nutrition in the ICU reduced the risk of delirium to some extent. Studies have shown that early rehabilitation training can reduce the incidence of delirium in mechanically ventilated ICU patients.^[24]^ This is because early rehabilitation training can increase the communication opportunities between healthcare personnel and patients, improve patients’ cognition, and thus reduce the occurrence of delirium; at the same time, early rehabilitation can help improve the quality of patients’ sleep at night by increasing the amount of patients’ daytime activities, promote the normalization of patients’ biological clocks, and thus reduce the risk of delirium; on the other hand, early rehabilitation training can improve the excitability of muscular neurons and promote axon growth in the compensatory cycle. Early rehabilitation training can enhance the excitability of muscle neurons and promote axonal growth in the compensatory cycle, stimulating the lateral cerebral hemisphere to achieve functional compensation and further improve the delirium situation rapidly. Some studies have shown that malnutrition in ICU patients is also a factor in delirium and its exacerbation and that early enteral nutrition support can significantly reduce the likelihood of delirium.^[25]^ Early enteral nutrition support is one of the nutritional pathways for mechanically ventilated patients that can meet the body’s caloric needs and maintain brain function while reducing oxidative stress and inflammatory response and regulating immune function. In conclusion, the combined implementation of analgesia and sedation, early rehabilitation, and early enteral nutrition reduces the incidence of delirium in mechanically ventilated patients.

### 4.3. Multidisciplinary analgesic and sedation intensive care can reduce the use of physical restraints in patients with tracheal intubation

The results of this study show that the use of physical restraints is lower in the intervention group than in the control group, suggesting that analgesic sedation cluster care based on multidisciplinary collaboration can reduce the use of physical restraints in patients with tracheal intubation. Patients with tracheal intubation usually have multiple tubes at the same time, such as central venous catheters and gastric tubes, and are often physically restrained to prevent unplanned extubation and other nursing safety events. Physical restraint is a common medical intervention in ICUs, with use rates ranging from 39.9% to 84.7%, and is useful in controlling delirious and agitated patient behavior, protecting patient safety, and preventing unplanned extubation, falls from bed, and self-harm. However, irrational physical restraints can cause psychological and physiological complications in patients, leading to an increase in the rate of unplanned extubation, etc.^[26]^ In addition, excessive use of physical restraint may also lead to skin breakdown at the site of restraint, peripheral circulation disorders in the limbs, compression of nerves in the limbs resulting in sensory abnormalities, limb movement dysfunction, and even medical disputes, so measures should be taken to reduce the use of excessive physical restraint. In this study, the implementation of analgesic sedation clustering measures ensured the rationality of analgesic sedation, avoided delirium and irritability caused by insufficient sedation, and reduced the use of physical restraint on patients. In physical restraint, the nurse is in charge of a 2-hour dynamic assessment for analgesia and sedation up to the standard of the patient’s timely release of restraint to avoid excessive restraint. Second, the psychological care in the ICU achieved the role of non-pharmacological analgesia and sedation through the psychological counseling of the counselor, the video visit of family members, playing of soothing music, and other measures, to give patients love and encouragement, to alleviate the patient’s anxiety, tension and other negative emotions, to achieve the effect of supporting the analgesia and sedation therapy to a certain extent to reduce the rate of use of physical restraints.

### 4.4. Multidisciplinary collaborative analgesic and sedation intensive care can improve cough strength and reduce mechanical ventilation time in tracheal intubated patients

The results of this study show that the cough strength of patients in the intervention group was improved compared with that of the control group. In contrast, the mechanical ventilation time of patients in the intervention group was shortened compared with that of the control group. The differences were statistically significant, indicating that multidisciplinary collaborative analgesic, sedative, and intensive care can improve the cough strength of patients with tracheal intubation and shorten mechanical ventilation time. The analgesic drug remifentanil has the advantages of fast onset of analgesia, strong effect, metabolism is not affected by liver and kidney function, but also has the advantages of short maintenance time, causing respiratory depression, skeletal muscle tonus, nausea and vomiting, hypotension and bradycardia and other side effects, so the use of remifentanil will lead to patients with cough and cough ability to be weakened. In the multidisciplinary ICU, the attending physician, respiratory therapist, and nurse in charge performed daily arousal and spontaneous breathing tests to determine the time of patient deactivation accurately and to reduce the use of medication due to delayed deactivation, thus reducing the inhibitory effect of drugs on the patient’s cough and sputum production. The ability of patients to cough up sputum to some extent influences the time of early extubation, and mechanical ventilation time is correspondingly longer in patients with poor coughing ability.^[27]^ Mechanical ventilation time is related to the patient’s ability to cough and various factors such as early activity^[28]^ and early feeding.^[29]^ In this study, early rehabilitation and early enteral nutrition were actively implemented to ensure the stability of ventilatory function and hemodynamic indices in mechanically ventilated patients through multidisciplinary intensive care, which emphasizes the cooperation and collaboration of multidisciplinary disciplines involving competent medical care, respiratory therapists, rehabilitation therapists, and nutrition therapists. Early rehabilitation improves the function of the patient’s respiratory and whole-body muscles through muscle and respiratory training that moves from passive to active activities, improves breathing, helps to increase the patient’s cough strength, reduces the patient’s dependence on the ventilator, and shortens the duration of mechanical ventilation in patients with tracheal intubation. In addition, early enteral nutrition ensured patients’ nutritional status, provided nutritional support for rehabilitation exercise and early weaning, and thus reduced the time on mechanical ventilation.

## 5. Conclusion

In conclusion, multidisciplinary analgesic and sedative bundled care has demonstrated the potential to decrease restraint use in patients undergoing endotracheal intubation and mechanical ventilation, reduce the need for analgesic and sedative medications, enhance cough strength, shorten the duration of mechanical ventilation, and improve overall patient experience and clinical outcomes. However, the study presents several limitations: the small sample size, focusing only on mechanically ventilated patients; a lack of objective measures and assessments of neurological recovery; and the brief intervention period, with no evaluation of long-term effects. Future research should expand the sample size, incorporate more objective indicators and neurological recovery assessments, and include long-term follow-up to assess sustained intervention outcomes better.

## Author contributions

**Conceptualization:** Xiaohui Wu, Longping Yu.

**Methodology:** Xiaohui Wu, Longping Yu.

**Writing—original draft:** Xiaohui Wu.

**Writing—review & editing:** Xiaohui Wu, Longping Yu.

**Formal analysis:** Longping Yu.

**Project administration:** Longping Yu.
